# Chicken *Enterococcus faecalis-*induced immunoglobulin Y as a prophylactic and therapeutic agent against streptococcosis in red tilapia (*Oreochromis* hybrid)

**DOI:** 10.14202/vetworld.2023.175-186

**Published:** 2023-01-28

**Authors:** Rifky Rizkiantino, Fachriyan Hasmi Pasaribu, Retno Damajanti Soejoedono, Wyanda Arnafia, Dinda Reisinta, Rifaldi Iqbal Yadiansyah, Beni Halalludin, Yunita Ardini, Granita Khanaria, I Wayan Teguh Wibawan

**Affiliations:** 1 Division of Medical Microbiology, Department of Infectious Animal Diseases and Veterinary Public Health, School of Veterinary Medicine and Biomedical Sciences, IPB University, Bogor, Indonesia; 2 Department of Central Laboratory, Division of Central Laboratory and Disease Research Center, Technology and Research Development, Central Proteina Prima (CP Prima) Inc., Tangerang, Indonesia; 3 Division of Research and Development, Tekad Mandiri Citra Co., Bandung, Indonesia; 4 Undergraduate Program of Applied Biology, Department of Biology, Faculty of Mathematics and Natural Sciences, University of Lampung, Bandar Lampung, Indonesia

**Keywords:** *Enterococcus faecalis*, immunoglobulin Y, immunoprophylaxis, immunotherapy, red tilapia, streptococcal infection

## Abstract

**Background and Aim:**

Streptococcosis is a common bacterial disease in red tilapia, in which *Enterococcus faecalis* infection has not been widely reported. This study aimed to evaluate the efficacy of pellets that contain chicken *E. faecalis*-induced immunoglobulin Y (IgY) to treat and prevent streptococcosis in red tilapia.

**Materials and Methods:**

We conducted a 28-day study for immunoprophylaxis and immunotherapy, each using four groups with two replications: Healthy control fish (KS), non-IgY pellets (PA and TA), pellets with 25% egg yolk containing *E. faecalis*-induced IgY (PB and TB), and pellets with 50% egg yolk containing *E. faecalis*-induced IgY(PC and TC). Indirect enzyme-linked immunosorbent assay was performed on prototype pellets produced with an IgY suspension at 1.63 mg/mL as the standard optical density curve. For the immunoprophylaxis study, pellets of 3% of the average body weight of the experimental fish (0.50 g per fish per day) were given daily until day 14 before the challenge test with *E. faecalis* (2.1 × 10^9^ Colony-forming unit/mL peroral) on day 15. The data from the observation period on days 15–28 were analyzed. For the immunotherapy study, pellets of 3% of the average body weight (0.50 g per fish per day) were given daily for 21 days (days 8–28) 7 day spost-infection. The data from the immunotherapy study were collected during the observation period on days 8–28. Statistical analysis was performed on non-specific immune variables: Total leukocytes, monocytes, lymphocytes, neutrophils, phagocytic activity, and macrophage capacity; and the semi-quantitative distribution of melanomacrophage centers (MMCs) in the lymphoid organs, such as spleen and liver. Photomacrographic data were analyzed descriptively and qualitatively by comparing the healing process and clinical signs found between experiments in the immunotherapy study.

**Results:**

The pellet with 50% egg yolk with an IgY at 2.43 mg/g pellet, 3% of body weight once daily, was the best formula on experimental fish. The administration of this formulation can also increase non-specific immunity and the distribution of MMCs in the spleen and liver with a survival rate of 55% for 14 days of challenge period in the immunoprophylaxis study and 70% for 21 days of therapy period in the immunotherapy study.

**Conclusion:**

Immunoglobulin Y can be a prophylactic and therapeutic agent against streptococcal infections caused *E. faecalis* in red tilapia with an optimum dosage of 2.43 mg/g pellet.

## Introduction

Tilapia (*Oreochromis niloticus*) is a popular freshwater aquaculture commodity in Indonesia. Widiati *et al*. [1] stated that tilapia first entered from Taiwan into Indonesia in 1969. Tilapia became popular among Indonesian after being introduced in 1971, spreading to various provinces in Indonesia. The tilapia belongs to the kingdom Animalia, phylum Chordata, class Osteichthyes, order Perciformes, family Cichlidae, and genus *Oreochromis* [1]. Tilapia have various colors: Blackish silver, silver blue, silver red, and yellowish. Tilapia can grow up to 50 cm in length, and the males are smaller than the females. This freshwater fish can be found easily in tropical waters with an average temperature of 8°C–30°C, such as rivers, estuaries, and lakes. Red tilapia is a hybrid species derived from a cross between blue tilapia (*Oreochromis aureus*) and Mozambique tilapia (*Oreochromis mossambicus*). The maximum length for red tilapia is 38 cm, with a body weight of 4.3 kg. This tilapia can tolerate brackish water environments with temperatures between 13°C and 37°C [2]. Like other aquaculture commodities, tilapia are also susceptible to infectious diseases. Streptococcosis is a bacterial disease in fish, in which *Streptococcus agalactiae*, *Streptococcus iniae* [3, 4], *Lactococcus garvieae* [5], and *Enterococcus faecalis* [6, 7] are common streptococcal infectious bacteria in tilapia.

Vaccination is a leading strategy to prevent streptococcosis in a cultural environment. Several studies have been conducted in developing streptococcal bacterial vaccines derived from field isolates. Wang *et al*. [8] reported the potential cross-immune reactions in Nile tilapia vaccinated with *S. agalactiae* and *S. iniae*. However, antibiotics were widely used to treat and control this disease. The use of antibiotics without adequate monitoring can trigger resistance, resulting in more challenges in treating this disease. Therefore, there is a need to explore the other potential materials that could be used to prevent and treat streptococcosis in tilapia.

Immunoglobulin Y (IgY) produced in chicken eggs has recently become a choice for the prophylactic or therapeutic agent in several diseases in fish. Li *et al*. [9] reported that injection of specific IgY against *Aeromonas hydrophila* at 30 mg/mL intraperitoneally (IP) could prevent bacterial infection by 60% and treat disease by 30% of the experimental fish. In Indonesia, field studies have also been reported on IgY as a potential anti-streptococcal infection caused by *S. agalactiae*. Mufidah *et al*. [10] claimed that IgY anti-*S. agalactiae* pellets given orally twice a week could prevent infection with a survival rate (SR) of 6.50%–6.75%. However, IgY concentrations and pellet formulations for prophylactic and therapeutic purposes through feed have not been explored in detail. Therefore, preventive and therapeutic agents against streptococcosis in red tilapia should be investigated. It is also essential to improve the packaging of IgY-contained pellets for optimal utilization of the IgY in experimental fish.

This study aimed to evaluate the efficacy of pellets containing IgY against *E. faecalis* in red tilapia. The optimal dose of IgY in pellets for prophylaxis and therapy was investigated to formulate the guideline for preventing and treating streptococcal infection.

## Materials and Methods

### Ethical approval

The Animal Ethics Committee, School of Veterinary Medicine and Biomedical Sciences, IPB University, Bogor, Indonesia, approved the use of experimental fish in this study on May 24, 2021, with the ethical approval certificate number 010/KEH/SKE/V/2021.

### Study period and location

The study was conducted with a total duration of 49 days (13-07-2021 to 30-08-2021), of which 21 days were for the quarantine and acclimatization period of the experimental fish and 28 days for the study period. The study was located at the Microbiology Laboratory and Aquatic Animal Health Laboratory, School of Veterinary Medicine and Biomedical Sciences, IPB University, Bogor, Indonesia; Division of Research and Development Laboratory, Tekad Mandiri Citra Co., Bandung, Indonesia; and Pathology and Histopathology Laboratory, Department of Central Laboratory, Division of Central Laboratory and Disease Research Center, Technology and Research Development, Central Proteina Prima (CP Prima) Inc., Tangerang, Indonesia.

### Formulation of the pellet containing *E. faecalis*-induced IgY

The whole egg yolks containing *E. faecalis*-induced IgY [11] and commercial fish pellets (HI-PRO-VITE 781 N, Central Panganpertiwi Animal Feedmill, Co., Ltd., Karawang, West Java, Indonesia) were previously evaluated for proximate analysis with two replications for determining the nutritional content. The whole egg yolk of 1 g was purified as described by Wibawan *et al*. [12]. The indirect enzyme-linked immunosorbent assay (ELISA) method was used to measure the *E. faecalis*-induced IgY using the IgY suspension concentration of 1.63 mg/mL as the standard optical density curve [13]. We performed the indirect ELISA with two replications before formulating the pellet mixture. After the commercial fish pellets were ground with a food mixer, whole egg yolk containing *E. faecalis*-induced IgY was added with the ratio of egg yolk containing *E. faecalis*-induced IgY: commercial feed as follows: 25% (w/w) was 100 g: 400 g and 50% (w/w) is 250 g: 250 g. To each mixture, 50 mL of phosphate buffer saline (PBS; Merck KGaA, Darmstadt, Germany) and 30 mL of encapsulation solution consisting of sodium alginate solution and 1% chitosan solution in a 1:1 were added gently and further homogenized using a food mixer. After being homogeneous, the feed mixture was milled and remolded using a pellet mill of ±2 mm. The pellets were put in the oven at 80°C for 30 min. Pellets containing *E. faecalis*-induced IgY with whole egg yolk concentrations of 25% and 50% were ready to use. The concentration of IgY in the pellets was again measured using indirect ELISA with two replications by sampling 1 g of pellet for each pellet prototype to make the extract. Proximate analysis with two replications was also conducted on the pellets prototype. The pellets were stored at 4°C and in a dry place until used in the efficacy test.

### Efficacy of the pellets containing *E. faecalis*-induced IgY as immunoprophylaxis and immunotherapy

We conducted a laboratory-based immunoprophylaxis study on healthy fish previously given pellets with a mixture of whole egg yolks containing *E. faecalis*-induced IgY at two different concentrations (25% and 50%). We used ten red tilapias (*Oreochromis* hybrid) with an average body weight of 16.70 ± 3.07 g in each experimental group with two replications. The experimental groups in this study were PA: The fish group that received only commercial pellets without *E. faecalis*-induced IgY, PB: Pellets with a mixture of 25% whole egg yolks containing *E. faecalis*-induced IgY, and PC: Pellets with a mixture of 50% whole egg yolks containing *E. faecalis*-induced IgY. Fish were quarantined and acclimatized for 21 days before the study.

Pellets were given daily at 3% of the average body weight of the experimental fish (0.50 g per fish per day) until the day 14 before the challenge test with *E. faecalis* strain 7INB [7] on day 15. Fish were also given non-IgY pellets of 3% body weight during the study period twice daily. Fish were challenged with a bacterial concentration of 2.1 × 10^9^ Colony-forming unit (CFU)/mL or equivalent to a standard McFarland 7 in 1 mL brain-heart infusion (BHI; Himedia, India) broth peroral (PO) in PA, PB, and PC experimental groups.

The laboratory-based immunotherapy study was conducted on healthy fish after 7 days challenging the manifestation of clinical signs [7]. We used ten red tilapias (*Oreochromis* hybrid) with an average body weight of 16.70 ± 3.07 g in each experiment with two replications. The experimental group in this study was TA: The fish group that received only commercial pellets without *E. faecalis*-induced IgY; TB: The fish group that received a mixture of 25% egg yolk containing *E. faecalis*-induced IgY; and TC: The fish group that received the pellets with a mixture of 50% egg yolk containing *E. faecalis*-induced IgY. Fish were also quarantined and acclimatized for 21 days before the study. Fish were challenged first on day 1 with *E. faecalis* strain 7INB [7] of 2.1 × 10^8^ CFU/mL in 1 mL BHI (Himedia) broth media (PO) in all experimental groups. Pellets were given daily for 21 days, at 3% of the average body weight of the experimental fish, that is, 0.50 g per fish per day, starting on the day 8 after the challenge test, and the fish started manifesting clinical signs. Fish were also given non-IgY pellets of 3% body weight during the study period twice daily.

The control group included ten healthy fish that received commercial pellets without containing *E. faecalis*-induced IgY. All experimental fish were observed until day 28. The experimental fish were kept in a solitary aquarium with dimensions of 15 × 15 × 18 cm with aeration and weekly water quality measurements, including temperature, total dissolved solids (TDS), pH, total ammonia nitrogen, and dissolved oxygen, were performed.

### Data collection

We collected the experimental fish’s initial and final body weight, total mortality, SR, and non-specific immune system variables such as total leukocytes, monocytes, lymphocytes, neutrophils, percentage of phagocytic activity, and macrophage capacity. The SR was calculated as described by Ahmadi *et al*. [14] as follows:


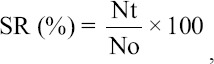


Where SR is the survival rate, No is the average number of fish at the beginning of the study period, and Nt is the average number of fish at the end of the study period.

Photomacrographs in the immunotherapy study were also collected to compare the clinical signs and to see the time-lapse of the healing process of clinical signs found in each experimental group.

Total and differential leukocytes count in fish was conducted using the Rosenfeld method [15] with three replications. The blood of 0.2–0.3 mL per fish was collected through the caudal vein and kept in a lithium heparin microtube 0.5 mL (Zhejiang Gongdong Medical Technology Co., Ltd., China). The blood was diluted with 0.65% NaCl solution with 1% crystal violet (Merck) using a leukocyte pipette (Superior Marienfeld, Paul Marienfeld GmbH & Co. KG, Königshofen, Germany). The diluted blood was counted for leukocytes using a Neubauer hemocytometer (MC, China). Leukocyte differentiation was performed by making blood smears fixed with 98% methanol, stained with 10% Giemsa staining (Merck), and then counted per 100 cells to obtain the relative values of monocytes, lymphocytes, and neutrophils [16].

The percentage of phagocytic activity and macrophage capacity was conducted by injecting the experimental fish with 0.5 mL of the *E. faecalis* suspension of 2.1 × 10^9^ CFU/mL or equivalent to a standard McFarland 7 intracoelomic (IC) and then incubating for 60 min. The experimental fish were euthanized using the low-temperature immersion method using ice water for 15 min until the fish reached the medullary collapse stage [17]. The fish were necropsied and washed in the coelomic cavity using a sterile 0.65% NaCl solution. The liquid was then collected in a 1.5 mL microtube for smearing, fixed using 98% methanol, and stained with 10% Giemsa (Merck). The percentage of phagocytic activity and macrophage capacity was calculated with three replications using the following formula [18]:


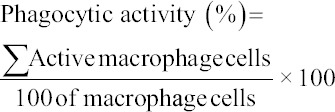



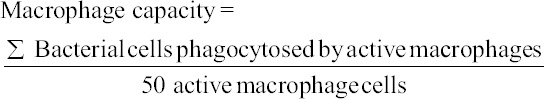


### Distribution of melanomacrophage centers (MMCs) in lymphoid organs

The histopathological examination was carried out to assess the MMCs using fish’s liver and spleen organs in each experimental group. Organ samples were fixed in formalin PBS (Merck) for trimming and then put in a tissue cassette. The samples were then dehydrated with an automatic tissue processing machine (Tissue-Tek VIP® 5 Jr, Sakura Finetek Japan Co. Ltd, Japan), embedded in liquid paraffin using an embedding machine (Tissue-Tek TEC™, Sakura Finetek Japan Co. Ltd), and cut to a thickness of ±5 mm using a rotary microtome (Accu-Cut^®^ SRM™, Sakura Finetek Japan Co. Ltd.). Then, the tissue samples were stained using hematoxylin-eosin (Merck) following Bancroft and Gamble method [19] with modifications.

The histopathological samples were semi-quantitatively examined by measuring the average MMCs surface area (%) in the spleen and liver (modification from Evans and Nowak [20]) by manually filtering the unique primary colors (current study: blue) using Adobe Photoshop CS5 software (Adobe Inc., California, USA) and quantified using ImageJ 64-bit Java 1.8.0_172 software (National Institutes of Health and the Laboratory for Optical and Computational Instrumentation, University of Wisconsin, Madison, USA). The percentage of MMCs surface area (%) was calculated using the following formula:


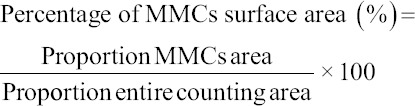


### Statistical analysis

Non-specific immune variables such as total leukocytes, monocytes, lymphocytes, neutrophils, percentage of phagocytic activity, and macrophage capacity were analyzed using analysis of variance at a 95% confidence interval. If data were significantly different, Duncan’s test was calculated with a 5% significance level. The melanomacrophage centers (MMCs) was analyzed semi-quantitative analysis by counting the percentage area of MMCs. Photomacrographic data were analyzed descriptively and qualitatively by comparing the healing process and clinical signs found between experiments in the immunotherapy study.

## Results

### Pellets formulation containing *E. faecalis*-induced IgY

Healthy fish swim up and down the tank and respond well to pellets. Pellets were given individually and little by little until the fish ate all the pellets according to the daily dose. Based on observations, increased turbidity was noted after administering pellets containing whole egg yolk IgY and a slightly oily effect on the aquarium walls the next day. However, it does not affect the swimming or feeding behavior of the fish due to periodic water changes. Moreover, the estimated concentration of IgY against *E. faecalis* contained in whole egg yolks, pellets with 25% egg yolk, and pellets with 50% egg yolk obtained 1.45 mg/g, 1.56 mg/g, and 2.43 mg/g, respectively. Proximate analysis showed that adding whole egg yolks containing *E. faecalis*-induced IgY, either in a ratio of 25% or 50%, did not cause changes in protein, ash, carbohydrates and crude fiber composition in pellets (Table-1). However, the presence of whole egg yolks was found to increase total fat content and energy from fat.

**Table-1:** Results of the proximate analysis of egg yolk and pellet prototype produced.

Nutrient content (unit)/100 g samples	Samples			

	A	B	C	D
Protein (%)	15.35 ± 0.16	27.71 ± 0.19	26.57 ± 0.15	27.23 ± 0.15
Ash (%)	2.51 ± 0.01	7.58 ± 0.04	6.65 ± 0.06	6.31 ± 0.11
Total fat (%)	27.60 ± 0.08	4.99 ± 0.01	10.38 ± 0.05	15.86 ± 0.05
Water content (%)	45.64 ± 0.02	9.36 ± 0.04	14.19 ± 0.07	13.70 ± 0.18
Total energy (kcal)	345.40 ± 0.41	357.21 ± 0.07	368.56 ± 0.27	399.26 ± 0.03
Carbohydrates (%)	8.92 ± 0.07	50.37 ± 0.20	42.22 ± 0.19	36.90 ± 0.03
Crude fiber (%)	1.05 ± 0.01	2.81 ± 0.02	3.39 ± 0.04	4.28 ± 0.02
Energy from fat (kcal)	248.36 ± 0.77	44.91 ± 0.09	93.42 ± 0.45	142.74 ± 0.45

The data are presented as Mean ± SD (n = 2). IgY = Immunoglobulin Y, A=Whole egg yolks contain IgY, B=Commercial pellet base, C=Commercial pellet base + 25% egg yolk containing IgY, D=Commercial pellet base + 50% egg yolk containing IgY

### Efficacy of *E. faecalis*-induced IgY as immunoprophylaxis and immunotherapy

**Figure-1:**
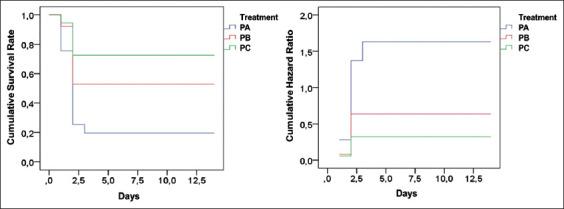
Graph of cumulative survival rate and hazard ratio in the immunoprophylaxis study based on the *E. faecalis*-induced IgY. IgY=Immunoglobulin Y, PA=Prophylaxis (non-IgY pellets), PB=Prophylaxis (25% IgY Pellets), PC=Prophylaxis (50% IgY Pellets).

The SR of fish that received non-IgY pellets, pellets with 25% egg yolk containing *E. faecalis*-induced IgY, and pellets with 50% egg yolk containing *E. faecalis*-induced IgY were 35%, 45%, and 55%, respectively. The cumulative SR and hazard ratio due to infection in the immunoprophylaxis study are presented in Figure-1. The group of fish treated with non-IgY pellets had the lowest survival. The mortality was observed for two days post-infection in all groups. In contrast, the fish group treated with pellets of 50% egg yolk containing *E. faecalis*-induced IgY (2.43 mg/g pellet) had the highest SR, with the stabilized incidence of mortality on day 2 of the challenge test.

**Figure-2:**
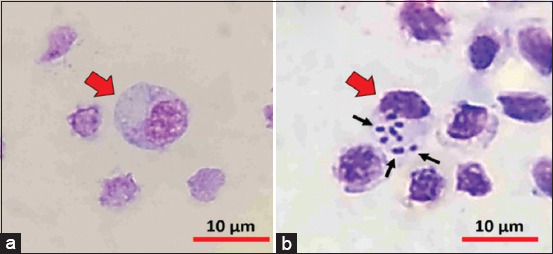
Photomicrograph visualization of phagocytic activity in macrophages of red tilapia (*Oreochromis* hybrid). (a) Inactive macrophages phagocytize bacteria (red arrow). (b) Active macrophages (red arrows) phagocytose *Enterococcus faecalis* (black arrows). Objective lens magnification = 100×.

Analysis of non-specific immune variables showed that pellets with 50% egg yolk containing *E. faecalis*-induced IgY (2.43 mg/g) significantly increased the non-specific immune system variable (p < 0.05) when compared with the control and other experimental groups in immunoprophylaxis and immunotherapy studies. However, in the immunoprophylactic study, the lymphocyte did not show any significant changes in the four groups of experimental fish; the results are presented in Tables-2 and 3, the visualization of macrophage activity is presented in Figure-2. In contrast, in the immunotherapy study, total leukocytes had a significant increase in the experimental fish that received the pellet with 25% and 50% egg yolk containing *E. faecalis*-induced IgY (p < 0.05) when compared to control healthy fish (8–9-fold higher) and the group of experimental fish given non-IgY pellets (2-fold higher). The test results variables are presented in Tables-4 and 5.

**Table-2:** White blood cell profile of experimental fish in the *E. faecalis*-induced IgY immunoprophylaxis study.

Experimental group	Variable white blood cells			

	Total leukocytes (10^3^/µL)	Monocytes (10^3^/µL)	Lymphocytes (10^3^/µL)	Neutrophils (10^3^/µL)
KS	14.47 ± 2.82^a^	3.58 ± 1.55^a^	8.57 ± 2.62^a^	2.32 ± 1.12^a^
PA	27.13 ± 4.95^b^	10.82 ± 5.37^b^	13.05 ± 6.99^a^	3.19 ± 1.74^a^
PB	37.72 ± 6.36^c^	20.40 ± 7.23^c^	11.79 ± 8.05^a^	5.53 ± 4.02^b^
PC	43.02 ± 4.78^d^	25.88 ± 5.69^d^	9.72 ± 4.93^a^	7.42 ± 3.62^c^

The data are presented as Mean ± SD (n = 20). Different superscript letters in the same column showed a significant difference (p < 0.05). IgY=Immunoglobulin Y. KS=Healthy control. PA=Prophylaxis (non-IgY pellets), PB=Prophylaxis (25% IgY Pellet), PC=Prophylaxis (50% IgY Pellet) IgY.

**Table-3:** Profiles of phagocytic activity and macrophage capacity of experimental fish in the *E. faecalis*-induced IgY immunoprophylaxis study.

Experimental group	Phagocytic activity (%)	Macrophage capacity (Bacterial cells/active macrophage)
KS	13.38 ± 10.70^a^	6.87 ± 3.98^a^
PA	29.55 ± 9.83^b^	12.08 ± 5.41^b^
PB	52.52 ± 10.85^c^	13.12 ± 6.85^b^
PC	65.83 ± 8.69^d^	19.18 ± 6.24^c^

The data are presented as Mean ± SD (n = 20). Different superscript letters in the same column showed a significant difference (p < 0.05). KS=Healthy control, PA=Prophylaxis (non-IgY pellets), PB=Prophylaxis (25% IgY Pellet), PC=Prophylaxis (50% IgY Pellet), IgY=Immunoglobulin Y

**Table-4:** White blood cell profile of experimental fish in the *E. faecalis*-induced IgY immunotherapy study.

Experimental group	Variable white blood cells			

	Total leukocytes (10^3^/µL)	Monocytes (10^3^/µL)	Lymphocytes (10^3^/µL)	Neutrophils (10^3^/µL)
KS	14.47 ± 2.82^a^	3.58 ± 1.55^a^	8.57 ± 2.62^a^	2.32 ± 1.12^a^
TA	57.99 ± 23.21^b^	22.52 ± 14.59^b^	28.64 ± 18.58^b^	6.83 ± 3.84^a^
TB	115.95 ± 44.56^c^	50.92 ± 22.91^c^	45.66 ± 34.82^c^	19.19 ± 13.89^b^
TC	126.37 ± 53.08^c^	68.25 ± 30.44^d^	39.55 ± 24.95^bc^	18.49 ± 9.26^b^

The data are presented as Mean ± SD (n = 20). Different superscript letters in the same column showed a significant difference (p < 0.05). KS=Healthy control, TA=Therapy (non-IgY pellets), TB=Therapy (25% IgY Pellets), TC=Therapy (50% IgY Pellets), IgY=Immunoglobulin Y

**Table-5:** Profiles of phagocytic activity and macrophage capacity of experimental fish in the *E. faecalis*-induced IgY immunotherapy study.

Experimental group	Phagocytic activity (%)	Macrophage capacity (Bacterial cells/active macrophage)
KS	13.38 ± 10.70^a^	6.87 ± 3.98^a^
TA	36.38 ± 12.68^b^	15.24 ± 8.48^b^
TB	58.92 ± 12.66^c^	16.35 ± 572^b^
TC	67.83 ± 14.85^d^	23.41 ± 10.78^c^

The data are presented as Mean ± SD (n = 20). Different superscript letters in the same column showed a significant difference (p < 0.05). KS=Healthy control, TA=Therapy (non-IgY pellets), TB=Therapy (25% IgY Pellets), TC=Therapy (50% IgY Pellets), IgY=Immunoglobulin Y

**Figure-3:**
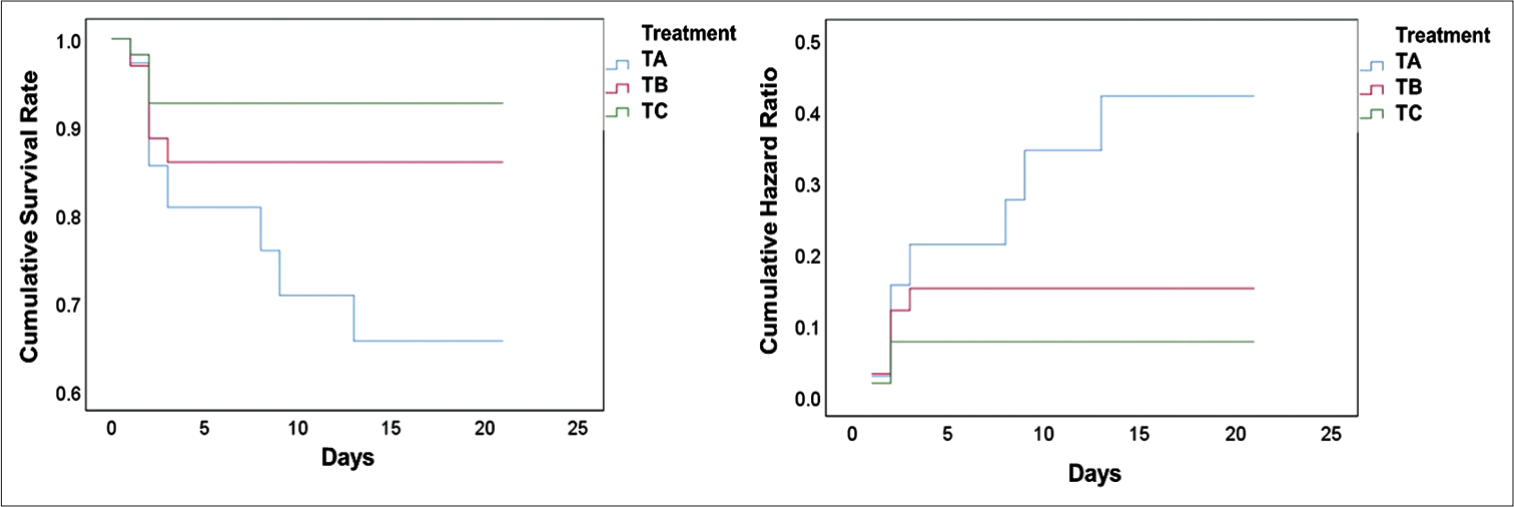
Graph of cumulative survival rate and hazard ratio in the immunotherapy study based on the *E. faecalis*-induced IgY. IgY = Immunoglobulin Y, TA=Therapy (non-IgY pellets), TB=Therapy (25% IgY Pellets), TC=Therapy (50% IgY Pellets).

The immunotherapy study showed the SR of the experimental fish groups that received non-IgY pellets, pellets with 25% egg yolk containing *E. faecalis*-induced IgY, and pellets with 50% egg yolk containing *E. faecalis*-induced IgY were 60%, 65%, and 70%, respectively. The cumulative survival and hazard level against streptococcal infection in the immunotherapy study are presented in Figure-3. The graph shows that the group of fish treated with non-IgY pellets had the lowest cumulative SR, in which the cumulative hazard could still be observed on the day 13 of observation. The group of fish treated with pellets with 50% egg yolk containing *E. faecalis*-induced IgY had the highest SR, with the last death incident on day 2 of the observation period.

**Figure-4:**
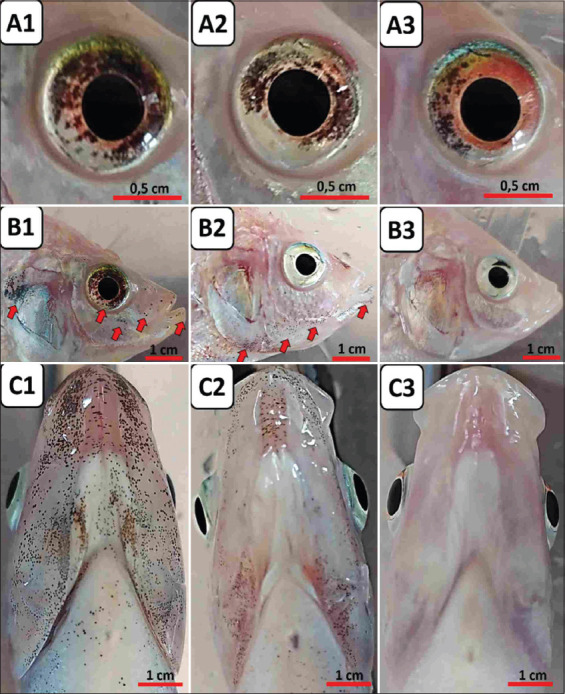
Comparison of clinical symptoms of streptococcal infection caused by *Enterococcus faecalis* on day 28 of an *E. faecalis*-induced IgY-based immunotherapy study. (a) Black spots and mild hyphema in the eye of the fish in the TA experimental group (1), the TB experimental group (2), and the TC experimental group (3). (b) Black spots on the operculum and mouth area of the experimental fish in the TA experimental group (1), the TB experimental group (2), and the TC experimental group (3). (c) Black spots on the ventral area of the gills and mouth on the experimental fish in the TA experimental group (1), the TB experimental group (2), and the TC experimental group (3). IgY = Immunoglobulin Y, TA=Therapy (non-IgY pellets), TB=Therapy (25% IgY Pellets), TC=Therapy (50% IgY Pellets).

The qualitative observations showed the therapeutic potential of IgY (Figure-4). Observations in the form of clinical signs of streptococcal infection due to *E. faecalis* represented as black spots on the eyes, operculum, mouth, and ventral gill area were observed for 21 days in the immunotherapy study. The clinical signs in the group of experimental fish given fed non-IgY pellets were more visible and significant compared to the other groups with IgY.

**Figure-5:**
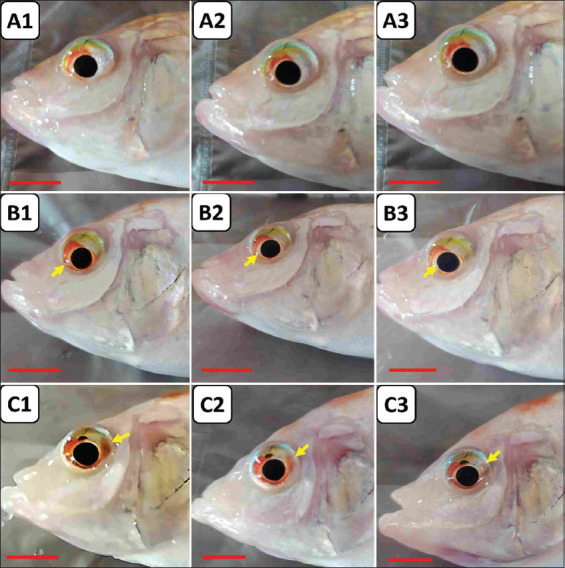
Photomacrograph of the therapy process for clinical symptoms of mild hyphema in the eye due to streptococcal infection caused by *Enterococcus faecalis* in the *E. faecalis*-induced IgY-based immunotherapy study. (a) Experimental fish in the TA experimental group on the 1^st^ (1), 7^th^ (2), and 14^th^ (3) days after pellet therapy was given. (b) The experimental fish of the TB experimental group on the 1^st^ (1), 7^th^ (2), and 14^th^ (3) days after the administration of therapeutic pellets. (c) Experimental fish in the TC experimental group on the 1^st^ (1), 7^th^ (2), and 14^th^ (3) days after pellet therapy was given. Scale bar=1 cm. IgY = Immunoglobulin Y, TA=Therapy (non-IgY pellets), TB=Therapy (25% IgY Pellets), TC=Therapy (50% IgY Pellets).

The healing process of clinical signs such as mild hyphema in the eyes was also observed until the day 14 of the immunotherapy study. Similarly, the healing process was more visible in the experimental fish given pellets with 25% and 50% egg yolk containing *E. faecalis*-induced IgY compared with the experimental fish of the non-IgY pellet group (Figure-5). Therefore, pellets with 25% and 50% egg yolk containing *E. faecalis*-induced IgY could provide a healing or therapeutic effect on mild hyphema in the eyes caused by *E. faecalis* infection. However, we still need to understand the underlying mechanism of IgY in reducing these clinical signs.

**Figure-6:**
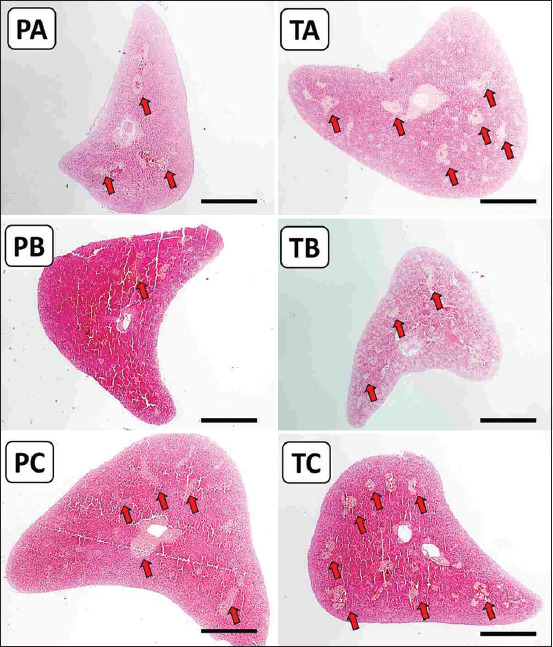
Photomicrograph of the surface area of melanomacrophage centers in the spleen (red arrow). PA: Prophylaxis (non-IgY pellets). PB: Prophylaxis (25% IgY Pellet). PC: Prophylaxis (50% IgY Pellet). TA: Therapy (non-IgY pellets). TB: Therapy (25% IgY Pellets). TC: Therapy (50% IgY Pellets). IgY=Immunoglobulin Y, Scale bar = 0.1 mm.

**Figure-7:**
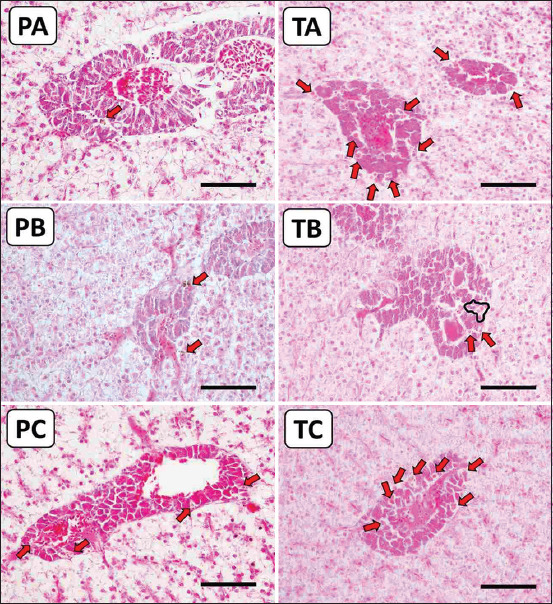
Photomicrograph of the melanomacrophage centers surface area in the liver organ (red arrows and black line area). PA: Prophylaxis (non-IgY pellets). PB: Prophylaxis (25% IgY Pellet). PC: Prophylaxis (50% IgY Pellet). TA: Therapy (non-IgY pellets). TB: Therapy (25% IgY Pellets). TC: Therapy (50% IgY Pellets). IgY=Immunoglobulin Y. Scale bar = 100 μm.

**Figure-8:**
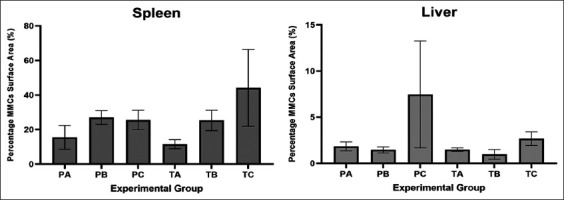
Percentage of the surface area of melanomacrophage centers (MMCs) in the spleen and liver. PA: Prophylaxis (non-IgY pellets). PB: Prophylaxis (25% IgY Pellet). PC: Prophylaxis (50% IgY Pellet). TA: Therapy (non-IgY pellets). TB: Therapy (25% IgY Pellets). TC: Therapy (50% IgY Pellets). IgY=Immunoglobulin Y.

Moreover, an increased MMCs surface area was observed in the fish of the 50% IgY group, both in immunoprophylaxis and immunotherapy studies (Figures-6 and 7). The spleen’s surface area of MMCs in the PA, PB, PC, TA, TB, and TC groups was 15.56%, 26.95%, 25.53%, 11.57%, 25.30%, and 44.08%, respectively (Figure-8). The same finding was found in the liver, where the surface area of MMCs in the PA, PB, PC, TA, TB, and TC groups was 1.83%, 1.46%, 7.47%, 1.48%, 0.97%, and 2.68%, respectively (Figure-8).

The water quality assessment during the 28-day study period confirmed the reasonable quality that could support the life of red tilapia in the immunoprophylaxis and immunotherapy studies (Table-6).

**Table-6:** Description of water quality (Mean ± SD) during the 28-day study period.

Experimental group	Water quality parameters				

	Temperature (°C)	pH	TDS (ppm)	Dissolved O_2_ (mg/L)	TAN (mg/L)
KS	26.85 ± 0.20	7.88 ± 0.12	91.70 ± 2.23	4.50 ± 1.76	0.02 ± 0.04
PA	26.60 ± 0.41	7.57 ± 0.44	71.91 ± 10.01	5.10 ± 0.04	0.02 ± 0.04
PB	26.50 ± 0.52	7.37 ± 0.40	65.41 ± 1.72	5.60 ± 1.53	0.00 ± 0.00
PC	26.60 ± 0.68	7.55 ± 0.32	66.00 ± 1.05	6.50 ± 2.32	0.00 ± 0.00
TA	26.70 ± 0.60	7.39 ± 0.21	67.68 ± 3.13	5.30 ± 1.12	0.00 ± 0.00
TB	26.59 ± 0.53	7.47 ± 0.26	74.84 ± 11.05	6.20 ± 1.75	0.06 ± 0.06
TC	26.40 ± 0.58	7.25 ± 0.26	68.80 ± 3.49	6.20 ± 1.12	0.01 ± 0.01

KS=Healthy control, PA=Prophylaxis (non-IgY pellets), PB=Prophylaxis (25% IgY Pellet), PC=Prophylaxis (50% IgY Pellet), TA=Therapy (non-IgY pellets), TB=Therapy (25% IgY Pellets), TC=Therapy (50% IgY Pellets), IgY=Immunoglobulin Y, TDS=Total dissolved solids, O_2_=Oxygen, TAN=Total ammonia nitrogen, SD=Standard deviation

## Discussion

Woolley and Landon [21] reported that IgY concentrations in an egg yolk ranged from 10 to 20 mg/mL and were 1.23-fold higher than serum IgY concentrations. The concentration obtained in this study was 1.45 mg/g of whole egg yolk, equivalent to 21.75 mg in an egg yolk. These results are close to the mean IgY concentration in egg yolk in the literature. Proximate analysis revealed that fat is needed as stored energy, signaling the body’s physiological regulation, and is required as a structural component to building cell membranes [22]. Moreover, Boujard *et al*. [23] found that in the diet-restricted group of *Dicentrarchus labrax*, the growth rate increased with increasing fat content in the feed. Physiologically, tilapia stores excess undigested lipids in adipose tissue through increased fatty acid absorption and triglyceride synthesis. Increasing the number but not increasing the size of adipose tissue is observed in tilapia due to continuous high-fat feeding [24]. Increased fat content and energy from fat in pellets can be an additional macronutrient for fish. Therefore, whole egg yolks containing specific IgY can provide extra nutrition with high-fat content and a therapeutic effect of specific IgY.

The findings in the immunopro­phylaxis study were consistent with similar studies for different bacterial species; Li *et al*. [9] reported that preventive measures by intraperitoneal administration with 30 mg/mL of the specific IgY against *A. hydrophila* at 1 × 10^9^ CFU/mL could protect the infection in 60% of *Carassius auratus Gibelio*. Zhang *et al*. [25] also reported that feeding supplemented with 10% egg yolk powder containing IgY anti-*Vibrio harveyi* showed a 50% SR in Japanese pufferfish (*Takifugu rubripes*), which was challenged with *V. harveyi* bacteria concentration of 5.4 × 10^7^ CFU/mL. Moreover, IgY using the immersion technique at 0.5 g/L showed an SR of 60%. It significantly increased the production of the enzymes such as superoxide dismutase, peroxidase, nitric oxide synthase, acid phosphatase, and lysozyme that play a role in the non-specific immune system in the experimental fish. Administration of IgY anti-*V. harveyi*, at 1 mg/mL, significantly increased macrophage phagocytosis in this study.

Phagocytosis by macrophages is one of the essential non-specific immune systems as the first gate in fighting bacterial infections and activating the specific immune system in the form of antibodies. Macrophages phagocytose bacteria and digest them into pieces of antigen which will then be presented to the surface through major histocompatibility complex (MHC) Class II proteins to helper T-cell receptors to stimulate the inflammatory process or antibody response. Then, activated B lymphocyte cells are facilitated by various cytokines, including interferon γ, interleukin 1, interleukin 2, interleukin 4, interleukin 5, and interleukin 13. Domain α1 and β1 in MHC Class II form MHC and antigen-binding regions. Activated B-cells mature and turn into plasma cells which later produce antibodies and develop memory cells to prevent reinfection in the future [26].

The use of IgY to treat bacterial infection in aquaculture is rarely reported. The results of this IgY-based immunotherapy study against *E. faecalis* indicated that IgY could be a therapeutic agent for streptococcosis in fish. Moreover, 7 days post-infection, pellets with 50% whole egg yolk containing *E. faecalis*-induced IgY (2.43 mg/g of pellets) reduced mortality by 30%. They maintained an average SR of 70% up to 21 days. Similar results have also been reported by Li *et al*. [9], which stated that the administration of IgY anti-*A. hydrophila* at 30 mg/mL IP 4 h post-infection by *A. hydrophila* can reduce the death incidence by 30% in *C. auratus* *Gibelio*. Similarly, Qin *et al*. [27] showed the administration of IgY anti-*A. hydrophila* injection at 5 mg per fish 12 h post-infection reduced mortality by 50% in *Megalobrama amblycephala*.

The use of antibiotics is associated with resistance to pathogenic bacteria, including *S. agalactiae* [28], *Edwardsiella tarda* [29, 30], *Vibrio* *parahaemolyticus* [31], *E. faecalis* [7], and *Aeromonas* spp. [32] in aquaculture. The antibiotic-resistance genes can spread to other bacteria in the aquatic and sedimentary environment [33]. This condition makes the need for more in-depth exploration of the use of different materials that not only have the potential as antibacterial but are also safe for the aquatic environment. The IgY protein, with its biological function as opsonin, agglutinin, precipitin, and inhibin, has the potential as an alternative therapy for bacterial diseases in aquaculture activities.

One of the functions of the antibodies or immunoglobulin is opsonin. Punt *et al*. [34] described opsonin as an extracellular protein that can induce phagocytic cells, such as macrophages and dendritic cells, to phagocytosed substances or cells. Opsonin provides markers for substances in the body, making it easy to carry out the cleansing process through phagocytic activity. These substances can be pathogens, such as bacteria, cancer cells, and protein-aggregates, such as amyloid. Phagocytosis by opsonin begins with the binding of opsonin to the target substance. Then, opsonin will bind to receptors of phagocytic cells on the other active site as liaison molecules between antigens and phagocytic cells. However, these protein molecules cannot bind to healthy cells or non-pathogenic substances due to pathogen-associated molecular patterns (PAMPs) that are specifically only possessed by pathogens or danger-associated molecular patterns in cancer cells. Phagocytic signals such as phosphatidylserine released by dead or stressed cells can also induce phagocytosis [35, 36].

At least 50 types of proteins are currently known as opsonins [35]. Complement proteins and antibodies are two types of proteins in the blood capable of opsonizing bacteria [37, 38]. In general, in the inflammatory process, bacteria-owned PAMPs can bind to receptors on phagocytic cells called pattern recognition receptors (PRRs). In addition to PRRs, phagocytic cells on their surface also express opsonin receptors, such as the fragment of complement (Fc) receptor on antibodies and the complement protein receptor C1. The coating of bacteria by opsonins in the form of antibodies on the fragment of antigen-binding will further stimulate the phagocytosis process conducted by phagocytic cells. The Fc part of the antibody will bind to the Fc receptor on phagocytic cells and cause antigen presentation to be better facilitated for phagocytosis. Furthermore, the more bacteria phagocytosed and eliminated, the more the specific adaptive immune system will be in the form of antibodies in the body [26, 39].

The increase in phagocytic activity and macrophage capacity causes an increased number of ingested bacterial cells. The IgY of the egg yolk origin is specific for *E. faecalis* and can act as a potential opsonin in red tilapia. An increase in the percentage of MMCs surface area in the spleen and liver organs of the PC and TC experimental groups also accompanied this increase.

MMCs are variables of the adaptive immune system in poikilothermic animals that can be measured quickly and cheaply. The MMCs are the pigmented phagocytic cells found in fish lymphoid organs, such as the cranial kidneys, spleen, and liver [40]. Studies have shown that when teleost is infected or under post-vaccination conditions, the MMCs in terms of surface area and cell count will be increased [41–44]. These findings are consistent with this study, which showed that the increase in MMCs in the group of fish given IgY was thought to occur due to the influence of the opsonization process, which increased phagocytic activity and the capacity of macrophages to ingest bacteria. The provision of ready-made antibody molecules can help stimulate the non-specific immune system in fish. The normal range for water quality variables in tilapia cultivation is temperature 25°C–32°C, pH 6.5–8.5, dissolved oxygen ≥3 mg/L [45], TAN for rearing red tilapia 0.17–3.87 mg/L [46], and TDS of <1000 ppm [47]. The water quality data showed that fish mortality was not related to poor water quality. This complementary data confirmed that the mortalities were not based on poor water quality.

## Conclusion

According to the proximate analysis, adding whole egg yolks can increase the total fat content and energy from fat in the pellets. The high-fat content can cause oily and cloudy water in the aquarium during the pelleting period. The pellet formulation of 50% egg yolk with an IgY concentration of 2.43 mg/g orally, with 3% body weight once a day, was the best in the experimental fish. The administration of this formulation can also increase non-specific immunity in the form of total leukocytes, monocytes, lymphocytes, neutrophils, phagocytic activity, macrophage capacity, and the distribution of MMCs in the spleen and liver with a SR of 55% for 14 days of challenge test and 70% for 21 days of therapy. The results indicated that IgY could be a prophylactic and therapeutic agent against streptococcal bacterial infections in red tilapia.

## Authors’ Contributions

RR: Conducted the immunological and microbiological examination of the specimen of *E. faecalis* and IgY anti-*E. faecalis*, the efficacy study in the experimental fish, and wrote the manuscript. IWTW, FHP, and RDS: Supervised microbiological and immunological examination. RIY: Managed the research data. WA and DR: Conducted to produce the chicken layer IgY. BH, YA, and GK: Conducted and advised in histopathological examination. All authors have read and approved the final manuscript.

## References

[1] Widiati A, Emmawati L, Hardjamulia A, Hardjamulia A (1999). Improving the genetic quality of fish through selection techniques. Proceedings of the Seminar on Fish Genetics Research Results;Time of Meeting Unknown (Year of Publication Unknown).

[2] Snow J (2015). Red Tilapia, *Oreochromis aureus* ×*Oreochromis mossambicus*.

[3] He R.Z, Li Z.C, Li S.Y, Li A.X (2021). Development of an immersion challenge model for *Streptococcus agalactiae* in Nile tilapia (*Oreochromis niloticus*). Aquaculture.

[4] Rahmatullah M, Ariff M, Kahieshesfandiari M, Daud H.M, Zamri-Saad M, Sabri M.Y, Amal M.N.A, Ina-Salwany M.Y (2017). Isolation and pathogenicity of *Streptococcus iniae* in cultured red hybrid tilapia in Malaysia. J. Aquat. Anim. Health.

[5] Bwalya P, Hang'ombe B.M, Evensen Ø, Mutoloki S (2021). *Lactococcus garvieae* isolated from Lake Kariba (Zambia) has low invasive potential in Nile tilapia (*Oreochromis niloticus*). J. Fish Dis.

[6] Rahman M, Rahman M.M, Deb S.C, Alam M.S, Alam M.J, Islam M.T (2017). Molecular identification of multiple antibiotic resistant fish pathogenic *Enterococcus faecalis* and their control by medicinal herbs. Sci. Rep.

[7] Rizkiantino R, Wibawan I.W.T, Pasaribu F.H, Soejoedono R.D, Arnafia W, Ulyama V, Wibowo D.B (2020). Isolation and characterisation of the *Enterococcus faecalis* strain isolated from red tilapia (*Oreochromis* hybrid) in Indonesia:A preliminary report. J. Surv. Fish. Sci.

[8] Wang Q, Fu T, Li X, Luo Q, Huang J, Sun Y, Wang X (2020). Cross-immunity in Nile tilapia vaccinated with *Streptococcus agalactiae* and *Streptococcus iniae* vaccines. Fish Shellfish Immunol.

[9] Li X.L, Shuai J.B, Fang W.H (2006). Protection of *Carassius auratus Gibelio* against infection by *Aeromonas hydrophila* using specific immunoglobulins from hen egg yolk. J. Zhejiang Univ. Sci. B.

[10] Mufidah T, Lusiastuti A.M, Purwaningsih U (2016). Field Test Using IgY Anti *Streptococcus agalactiae* for Immunotherapy of Streptococcosis in BEST Tilapia Culture.

[11] Rizkiantino R, Wibawan I.W.T, Pasaribu F.H, Soejoedono R.D, Poetri O.N, Arnafia W, Sasih K.D, Reisinta D (2020). The potential of adjuvant against production of antistreptococcal immunoglobulin Y (IgY) in aquaculture. Indones. J. Vet. Sci.

[12] Wibawan I.W.T, Kristanti N.D, Zulfa A, Sasi K.D, Permatasari D.A, Cahyono M.I, Julianto Sibit, G, Arnafia W (2018). Production of IgY against infectious bursal disease virus and purification of IgY from egg by using biocompatible technique. Intern. J. Appl. Res. Vet. Med.

[13] Rizkiantino R (2022). Immunoprophylaxis and Immunotherapy Based on Chicken Egg's Immunoglobulin Y (IgY) Against Streptococcal Infection in Red Tilapia (*Oreochromis* hybrid). [Dissertation].

[14] Ahmadi M.R, Mahmoudzadeh H, Babaei M, Mehrjand M.S (2011). Prediction of survival rate in European white fish (*Coregonus lavaretus*) fry on three different feeding regimes. Iran. J. Fish. Sci.

[15] Rosenfeld G (1947). Corante pancrômico para hematologia e citologia clínica. Nova combinação dos componentes do May-Grunwald e do Giemsa num sócorante de emprego rápido. Mem. Inst. Butantan.

[16] Rizkiantino R, Pasaribu F.H, Soejoedono R.D, Purnama S, Wibowo D.B, Wibawan I.W.T (2021). Experimental infection of *Enterococcus faecalis* in red tilapia (*Oreochromis* hybrid) revealed low pathogenicity to cause streptococcosis. Open Vet. J.

[17] Stoskopf M (2010). Fish Medicine.

[18] Atlas R.M (1984). Phagocytosis in Microbiology Fundamentals and Application.

[19] Bancroft J.D, Gamble M (2008). Theory and Practice of Histological Techniques.

[20] Evans D, Nowak B (2016). Effect of ranching time on melanomacrophage centres in anterior kidney and spleen of Southern bluefin tuna, *Thunnus maccoyii*. Fish Shellfish Immunol.

[21] Woolley J.A, Landon J (1995). Comparison of antibody production to human interleukin-6 (IL-6) by sheep and chickens. J. Immunol. Methods.

[22] Eckel R.H, Grundy S.M, Zimmet P.Z (2005). The metabolic syndrome. Lancet.

[23] Boujard T, Gélineau A, Covès D, Corraze G, Dutto G, Gasset E, Kaushik S (2004). Regulation of feed intake, growth, nutrient and energy utilisation in European sea bass (*Dicentrarchus labrax*) fed high fat diets. Aquaculture.

[24] He A.Y, Ning L.J, Chen L.Q, Chen Y.L, Xing Q, Li J.M, Qiao F, Li D.L, Zhang M.L, Du Z.Y (2015). Systemic adaptation of lipid metabolism in response to low-and high-fat diet in Nile tilapia (*Oreochromis niloticus*). Physiol. Rep.

[25] Zhang M, Geng H, Javed M.T, Xu L, Li X, Wang L, Li S, Xu Y (2021). Passive protection of Japanese pufferfish (*Takifugu rubripes*) against *Vibrio harveyi* infection using chicken egg yolk immunoglobulins (IgY). Aquaculture.

[26] Janeway C.A, Travers P, Walport M, Shlomchik M.J (2002). Immunobiology:The Immune System in Health and Disease.

[27] Qin Z, Babu V.S, Li N, Fu T, Li J, Yi L, Zhao L, Li J, Zhou Y, Lin L (2018). Protective effects of chicken egg yolk immunoglobulins (IgY) against experimental *Aeromonas hydrophila* infection in blunt snout bream (*Megalobrama amblycephala*). Fish Shellfish Immunol.

[28] Deng L, Li Y, Geng Y, Zheng L, Rehman T, Zhao R, Wang K, OuYang P, Chen D, Huang X, He C, Yang Z, Lai W (2019). Molecular serotyping and antimicrobial susceptibility of *Streptococcus agalactiae* isolated from fish in China. Aquaculture.

[29] Xiao J, Wang Q, Liu Q, Wang X, Liu H, Zhang Y (2009). Isolation and identification of fish pathogen *Edwardsiella tarda* from mariculture in China. Aquac. Res.

[30] Rizkiantino R, Wibawan I.W.T, Pasaribu F.H, Soejoedono R.D, Arnafia W, Ulyama V, Nugraha K.A (2021). Isolation and characterisation of *Edwardsiella tarda* in barred bichir fish (*Polypterus delhezi*, Boulenger 1899) with septicemia:A first case report. Vet. Pract.

[31] Tan C.W, Rukayadi Y, Hasan H, Thung T.Y, Lee E, Rollon W.D, Hara H, Kayali A.Y, Nishibuchi M, Radu S (2020). Prevalence and antibiotic resistance patterns of *Vibrio parahaemolyticus* isolated from different types of seafood in Selangor, Malaysia. Saudi J. Biol. Sci.

[32] Fauzi N.N.F.N.M, Hamdan R.H, Mohamed M, Ismail A, Mat Zin A.A, Mohamad N.F.A (2021). Prevalence, antibiotic susceptibility, and presence of drug resistance genes in *Aeromonas* spp. Isolated from freshwater fish in Kelantan and Terengganu states, Malaysia. Vet. World.

[33] Watts J.E.M, Schreier H.J, Lanska L, Hale M.S (2017). The rising tide of antimicrobial resistance in aquaculture:Sources, sinks and solutions. Mar. Drugs.

[34] Punt J, Stranford S.A, Jones P.P, Owen J.A (2019). Kuby Immunology.

[35] Escamilla-Tilch M, Filio-Rodríguez G, García-Rocha R, Mancilla-Herrera I, Mitchison N.A, Ruiz-Pacheco J.A, Sánchez-García F.J, Sandoval-Borrego D, Vázquez-Sánchez E.A (2013). The interplay between pathogen-associated and danger-associated molecular patterns:An inflammatory code in cancer?. Immunol. Cell Biol.

[36] Cockram T.O.J, Dundee J.M, Popescu A.S, Brown G.C (2021). The phagocytic code regulating phagocytosis of mammalian cells. Front. Immunol.

[37] Merle N.S, Noe R, Halbwachs-Mecarelli L, Fremeaux-Bacchi V, Roumenina L.T (2015). Complement system part II:Role in immunity. Front. Immunol.

[38] Chiu M.L, Goulet D.R, Teplyakov A, Gilliland G.L (2019). Antibody structure and function:The basis for engineering therapeutics. Antibodies (Basel).

[39] Parham P (2005). The Immune System.

[40] Agius C (1979). The role of melano-macrophage centres in iron storage in normal and diseased fish. J. Fish Dis.

[41] Steinel N.C, Bolnick D.I (2017). Melanomacrophage centers as a histological indicator of immune function in fish and other poikilotherms. Front. Immunol.

[42] Secombes C.J (1982). Histological changes in lymphoid organs of carp following injection of soluble or particulate antigens. Dev. Comp. Immunol.

[43] Vogelbein W.K, Fournie J.W (1987). Sequential development and morphology of experimentally induced hepatic melano-macrophage centres in *Rivulus marmoratus*. J. Fish Biol.

[44] Kranz H (1989). Changes in splenic melano-macrophage centers of dab *Limanda limanda* during and after infection with ulcer disease. Dis. Aquat. Organ.

[45] Cavalcante D.D.H, Caldini N.N, da Silva J.L.S, Lima F.R.D.S, Docarmo M.V (2014). Imbalance in the hardness/alkalinity ratio of water and Nile tilapia's growth performance. Acta Sci. Technol.

[46] Caldini N.N, Cavalcante D.D.H, Filho P.R.N.R, Docarmo M.V (2015). Feeding Nile tilapia with artificial diet and dried bioflocs biomass. Acta Sci. Anim. Sci.

[47] Boyd C.E, Massaut L (1999). Risks associated with the use of chemicals in pond aquaculture. Aquac. Eng.

